# Dataset on the importation of the exotic shrimp *Penaeus vannamei* broodstock (Boone, 1931) to India

**DOI:** 10.1016/j.dib.2017.02.034

**Published:** 2017-02-21

**Authors:** M.C. Remany, R. Kirubagaran, Daly Cyriac, P. Krishnakanth Varadaraju, Sruthi Prem O.C, A.K. Panda, Jaideep Kumar

**Affiliations:** a Department of Marine Biotechnology, Sathyabama University, Jeppiar Nagar, Chennai 600119, Tamil Nadu, India; b Aquatic Quarantine Facility for *L. vannamei*, Rajiv Gandhi Centre for Aquaculture, MPEDA (Ministry of Commerce and Industry, Government of India) TNFDC Hatchery Complex,Kapaleeswarar Nagar, Beach Road, Neelankarai, Chennai 600041, Tamil Nadu, India; c Marine Biotechnology Division, Ocean Science & Technology for Islands Group, National Institute of Ocean Technology (Ministry of Earth Sciences, Government of India) Pallikaranai, Chennai 600100, Tamil Nadu, India; d Rajiv Gandhi Centre for Aquaculture, Technology Transfer Training & Administrative Complex, MPEDA (Ministry of Commerce and Industry, Govt. of India), 3/197, Poompuhar Road, Karaimedu, Sattanathapuram, Sirkali, 609109 Nagapattinam District, Tamil Nadu, India

**Keywords:** Broodstock, Transit, Quarantine

## Abstract

*Penaeus vannamei* is an exotic shrimp species that has gained high culture momentum, since its introduction to India [1]. Currently, the culture of the species in the Country is being done by the shrimp farmers by importation of Specific Pathogen Free (SPF) vannamei broodstock from approved suppliers, which are located overseas. The value of one brooder normally ranges from 50 to 61 US $, excluding the custom duty, processing fee and other charges for the transboundary shipment of the stock to India. The *P. vannamei* stock are permitted to be imported to the Country by the hatchery operators only through the single declared port of entry, i.e. Chennai in Tamil Nadu in the Country. The imported parent shrimps are then to be quarantined at the Aquatic Quarantine Facility before being transported to the vannamei hatcheries [2]. This article reports the data available on import of vannamei broodstock to India since its importation to India in 2009. The dataset presented here contains information on transit and quarantine mortality of the brooders following the shipment of the stock by the various broodstock suppliers from the overseas.

**Specifications Table**

**Table:** 

Subject area	*Biology*
More specific subject area	*Aquaculture*
Type of data	*Table and graph*
How data was acquired	*Direct source from the Quarantine unit for Penaeus vannamei, the only one such facility in India*
Data format	*Analyzed*
Experimental factors	*Influence of shipment duration on transit mortality and quarantine survival of imported P. vannamei brooders*
Experimental features	*Shipment mortality and survival during quarantine*
Data source location	*India*
Data accessibility	*Public repository*

**Value of the data**

• The Aquatic Quarantine Facility (AQF) being the only dedicated Government approved quarantine centre for *P. vannamei*, the data generated from the facility is the only source of information on the entry of the exotic shrimp species into the Country.
• Benefits the aquaculture research and as well as the sector by providing traceability to the stock that is being produced from India.
• Data provides information on the quantum of broodstock and its survival when imported from overseas suppliers during its transboundary shipment and quarantine.
• Comparative analysis of the data presented here would help the suppliers to take appropriate measures to minimize the mortality of the highly valued brooders during shipment of the broodstock.
• Provides secondary information for the policy makers on the shrimp production of the Country based on the importation data.

## 1 Data

The data presented in this article shows the supplier wise import, quarantine and transit mortality of the *P. vannamei* stock since the introduction of vannamei culture in the Country (Table 1). A comparison of the mortality rates (percentage mean) during shipment and quarantine is provided in Table 2.

## 2 Experimental design, materials and methods

**Fig. 1:**
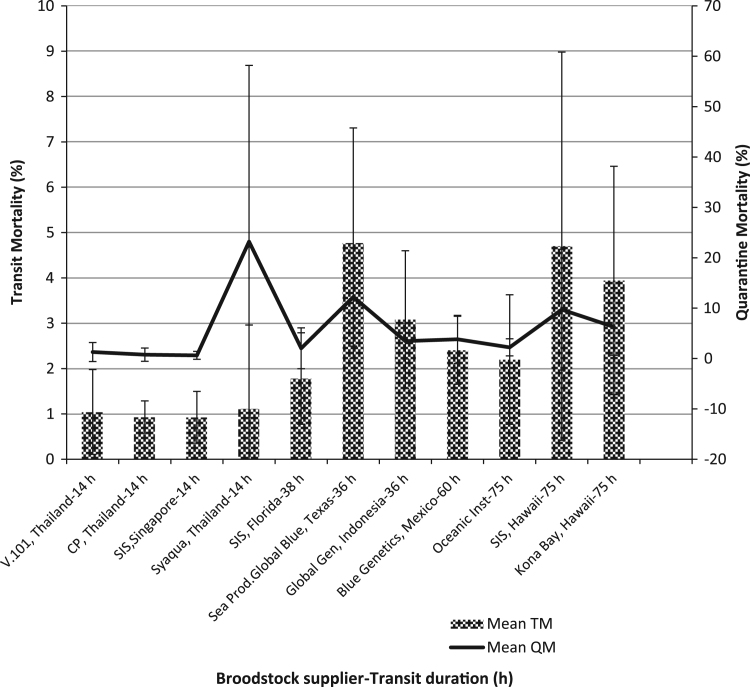
Relation between transit and quarantine mortalities (Mean±SEM) of *P. vananmei* broodstock supplied to India by different broodstock suppliers.

**Fig. 2:**
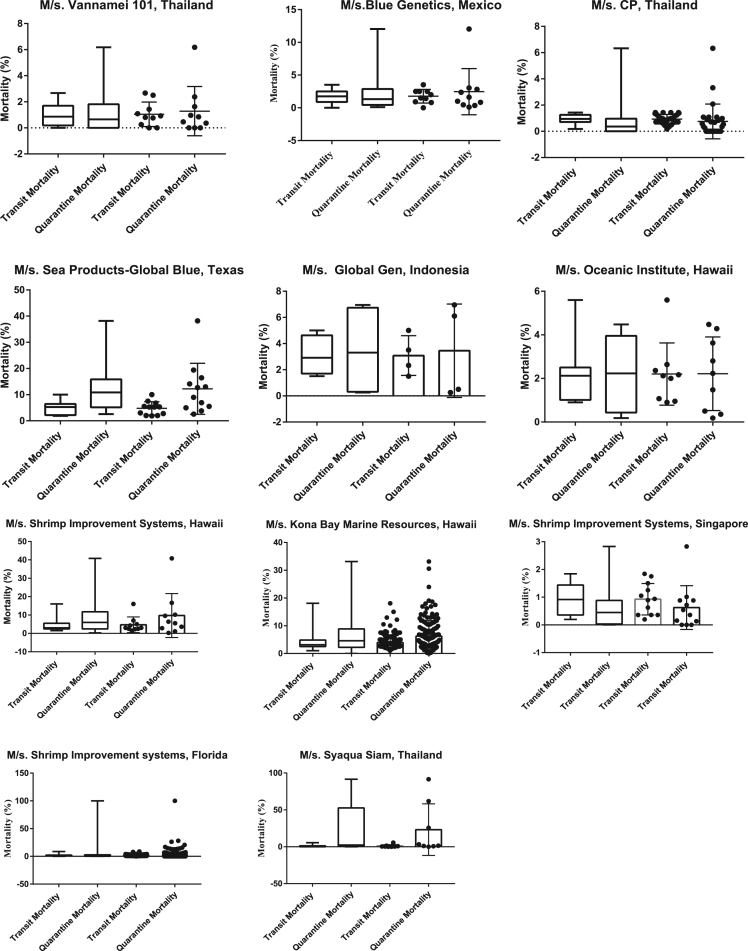
Scatter plot and box whisker representation of percentage mortalities of imported *P. vannamei* brooders.

The importation data of *P. vannamei* brooders to India from different approved broodstock suppliers was collected from the quarantine facility, since its inception. The data on transit and quarantine mortality was obtained and subjected to statistical analysis using Graphpad prism 7.0 software. The normal duration of broodstock shipment from the suppliers is provided. However, the unusual delays in shipment caused due to flight delays and change in flight routes are not considered while indicating the overall shipment duration in the data given. The mean and the standard error of the data when normally distributed is presented. Tukeys Multiple comparison test at 0.05 level was used to compare the data obtained on quarantine and shipment mortalities between the different suppliers (Fig. 1, Fig. 2).

## Figures and Tables

**Table 1:** Tukeys Multiple Comparison between *P. vannamei* broodstock suppliers on the basis of percentage of mean transit and quarantine mortalities.

**Comparison between supplier groups**	**Transit Mortality** **(%mean)**	**Quarantine Mortality(% mean)**
V.101, Thailand *vs*. Blue Genetics, Mexico	ns	ns
V.101, Thailand *vs*. CP, Thailand	ns	ns
V.101, Thailand *vs*. Global Blue, Texas	s	s
V.101, Thailand *vs*. Global Gen, Indonesia	ns	ns
V.101, Thailand *vs*. Oceanic Inst	ns	ns
V.101, Thailand *vs*. SIS, Hawaii	s	ns
V.101, Thailand *vs*. Kona Bay, Hawaii	s	ns
V.101, Thailand *vs*. SIS, Singapore	ns	ns
V.101, Thailand *vs*. SIS, Florida	ns	ns
V.101, Thailand *vs*. Syaqua, Thailand	ns	s
Blue Genetics, Mexico *vs*. CP, Thailand	ns	ns
Blue Genetics, Mexico *vs*. Global Blue, Texas	ns	ns
Blue Genetics, Mexico *vs*. Global Gen, Indonesia	ns	ns
Blue Genetics, Mexico *vs*. Oceanic Inst	ns	ns
Blue Genetics, Mexico *vs*. SIS, Hawaii	ns	ns
Blue Genetics, Mexico *vs*. Kona Bay, Hawaii	ns	ns
Blue Genetics, Mexico *vs*. SIS, Singapore	ns	ns
Blue Genetics, Mexico *vs*. SIS, Florida	ns	ns
Blue Genetics, Mexico *vs*. Syaqua, Thailand	ns	ns
CP, Thailand *vs*. Global Blue, Texas	s	s
CP, Thailand *vs*. Global Gen, Indonesia	ns	ns
CP, Thailand *vs*. Oceanic Inst	ns	ns
CP, Thailand *vs*. SIS, Hawaii	s	s
CP, Thailand *vs*. Kona Bay, Hawaii	s	s
CP, Thailand *vs*. SIS, Singapore	ns	ns
CP, Thailand *vs*. SIS, Florida	ns	ns
CP, Thailand *vs*. Syaqua, Thailand	ns	s
Global Blue, Texas *vs*. Global Gen, Indonesia	ns	ns
Global Blue, Texas *vs*. Oceanic Inst	s	s
Global Blue, Texas *vs*. SIS, Hawaii	ns	ns
Global Blue, Texas *vs*. Kona Bay, Hawaii	ns	s
Global Blue, Texas *vs*. SIS, Singapore	s	s
Global Blue, Texas *vs*. SIS, Florida	s	s
Global Blue, Texas *vs*. Syaqua, Thailand	s	s
Global Gen, Indonesia *vs*. Oceanic Inst	ns	ns
Global Gen, Indonesia *vs*. SIS, Hawaii	ns	ns
Global Gen, Indonesia *vs*. Kona Bay, Hawaii	ns	ns
Global Gen, Indonesia *vs*. SIS, Singapore	ns	ns
Global Gen, Indonesia *vs*. SIS, Florida	ns	ns
Global Gen, Indonesia *vs*. Syaqua, Thailand	ns	s
Oceanic Inst *vs*. SIS, Hawaii	s	ns
Oceanic Inst *vs*. Kona Bay, Hawaii	s	ns
Oceanic Inst *vs*. SIS, Singapore	ns	ns
Oceanic Inst *vs*. SIS, Florida	ns	ns
Oceanic Inst *vs*. Syaqua, Thailand	ns	s
SIS, Hawaii *vs*. Kona Bay, Hawaii	ns	ns
SIS, Hawaii *vs*. SIS, Singapore	s	ns
SIS, Hawaii *vs*. SIS, Florida	s	s
SIS, Hawaii *vs*. Syaqua, Thailand	s	s
Kona Bay, Hawaii *vs*. SIS, Singapore	s	ns
Kona Bay, Hawaii *vs*. SIS, Florida	s	s
Kona Bay, Hawaii *vs*. Syaqua, Thailand	s	s
SIS,Singapore *vs*. SIS, Florida	ns	ns
SIS,Singapore *vs*. Syaqua, Thailand	ns	s
SIS, Florida *vs*. Syaqua, Thailand	ns	s

s-significant; ns-not significant; level of significance-0.05.

**Table 2:** One way-ANOVA (α=0.05 level) test results on the influence of transit mortality on quarantine mortality of *P. vannamei* broodstock.

**S. No.**	**Broodstock Supplier**	**Calculated*****p*****value**
1	Vannamei 101, Thailand	0.724^a^
2	SIS, Singapore	0.295 a
3	Blue Genetics, Mexico	0.534 a
4	CP, Thailand	0.505 a
5	Sea Products-Global Blue Technologies, Texas	0.017^b^
6	Global Gen, Indonesia	0.856 a
7	Oceanic Institute, Hawaii	0.984 a
8	SIS, Hawaii	0.229 a
9	Syaqua Siam, Thailand	0.096 a
10	Kona Bay, Hawaii	0.004 a
11	SIS, Florida	0.057^b^

Values superscripted as

a indicates no significant effect.

b indicates significant effect.
