# Supplementary material for: Dataset on the importation of the exotic shrimp Penaeus vannamei broodstock (Boone, 1931) to India
Source: Data Brief. 2017 Feb 21;11:527–32. doi: 10.1016/j.dib.2017.02.034 (PMC5357676; doi:10.1016/j.dib.2017.02.034)
Supplement: Supplementary file 2 — Supplementary material [file mmc2.docx]

**Data on transit and quarantine mortalities of individual *P. vannamei* broodstock batches imported to India from broodstock suppliers**

1. From M/s. Vannamei 101 (van 101), Thailand (Transit duration-14 hrs) during the period 2009-2012

| *Total brooders supplied* | *Transit Mortality (%)* | *Quarantine Mortality (%)* |
| --- | --- | --- |
| 3192 | \| 1.09 \| \| --- \| \| 0.00 \| \| 2.67 \| \| 0.25 \| \| 0.76 \| \| 0.00 \| \| 1.43 \| \| 0.92 \| \| 0.80 \| \| 2.50 \| | \| 6.18 \| \| --- \| \| 0.96 \| \| 0.00 \| \| 0.00 \| \| 0.00 \| \| 0.85 \| \| 0.36 \| \| 0.46 \| \| 1.63 \| \| 2.38 \| |
|  |  |  |
| *Mean ±SE (n=10)* | 1.04 ± 0.29% | 1.23 ± 0.59% |

1. *From M/s. Syaqua Siam, Thailand* (Transit duration-14 hrs) during the period 2009-2013

| *Total brooders supplied* | *Transit Mortality (%)* | *Quarantine Mortality (%)* |
| --- | --- | --- |
| 3180 | \| 0.45 \| \| --- \| \| 0.80 \| \| 0.00 \| \| 0.36 \| \| 5.54 \| \| 1.43 \| \| 0.36 \| \| 0.00 \| | \| 61.76 \| \| --- \| \| 91.57 \| \| 1.38 \| \| 1.08 \| \| 25.69 \| \| 0.72 \| \| 3.32 \| \| 0.00 \| |
| *Mean ± SE (n=8)* | 1.12 ± 0.65% | 23.19 ± 12.37% |

*( c) From M/s. CP Aqua foods, Thailand* (Transit duration-14 hrs) during the period 2009-2013

| *Total brooders supplied* | *Transit Mortality (%)* | *Quarantine Mortality (%)* |
| --- | --- | --- |
| 14024 | \| 0.50 \| \| --- \| \| 0.67 \| \| 1.36 \| \| 0.78 \| \| 1.43 \| \| 0.46 \| \| 1.00 \| \| 0.18 \| \| 0.71 \| \| 0.95 \| \| 0.71 \| \| 0.71 \| \| 0.71 \| \| 1.07 \| \| 0.71 \| \| 0.36 \| \| 1.43 \| \| 1.43 \| \| 1.43 \| \| 1.00 \| \| 0.97 \| \| 1.39 \| \| 1.25 \| \| 1.18 \| \| 0.86 \| \| 0.94 \| \| 1.25 \| \| 0.63 \| | \| 0.25 \| \| --- \| \| 0.00 \| \| 2.08 \| \| 0.59 \| \| 0.00 \| \| 0.69 \| \| 0.00 \| \| 3.32 \| \| 1.08 \| \| 6.33 \| \| 0.00 \| \| 0.36 \| \| 1.08 \| \| 0.00 \| \| 1.08 \| \| 0.36 \| \| 0.00 \| \| 0.00 \| \| 0.54 \| \| 0.00 \| \| 0.98 \| \| 0.84 \| \| 0.00 \| \| 0.89 \| \| 0.72 \| \| 0.00 \| \| 0.00 \| \| 0.00 \| |
|  |  |  |
| *Mean ± SE (n=28)* | *0.93 ± 0.06%* | *0.76 ± 0.25%* |

1. *From M/s. Blue Genetics, California, Mexico* (Transit duration-60 hrs) during the period 2015-2016

| *Total brooders supplied* | *Transit Mortality (%)* | *Quarantine Mortality (%)* |
| --- | --- | --- |
| 3760 | \| 2.00 \| \| --- \| \| 1.50 \| \| 2.50 \| \| 2.50 \| \| 3.52 \| | \| 2.83 \| \| --- \| \| 12.04 \| \| 1.01 \| \| 3.04 \| \| 0.11 \| |
| *Mean ± SE (n=5)* | *2.41 ± 0.33%* | *3.81 ± 2.13%* |

*(e) From M/s. Sea Products Development-Global Blue, Texas, USA* (Transit duration-36 hrs) during the period 2015-2016

| *Total brooders supplied* | *Transit Mortality (%)* | *Quarantine Mortality (%)* |
| --- | --- | --- |
| 7100 | \| 2.75 \| \| --- \| \| 3.00 \| \| 10.00 \| \| 1.88 \| \| 2.00 \| \| 2.00 \| \| 5.33 \| \| 5.25 \| \| 6.75 \| \| 5.25 \| \| 7.50 \| \| 5.50 \| | \| 38.17 \| \| --- \| \| 6.95 \| \| 8.99 \| \| 16.45 \| \| 12.68 \| \| 12.99 \| \| 3.81 \| \| 4.99 \| \| 5.54 \| \| 19.40 \| \| 14.12 \| \| 2.56 \| |
| *Mean ± SE (n=12)* | *4.77 ± 0.73%* | *12.22 ± 2.82%* |

*(f) From M/s. Global Gen, Indonesia* (Transit duration-36 hrs) during the period 2014-2016

| *Total brooders supplied* | *Transit Mortality (%)* | *Quarantine Mortality (%)* |
| --- | --- | --- |
| 2400 | \| 3.50 \| \| --- \| \| 2.33 \| \| 5.00 \| \| 1.50 \| | \| 6.95 \| \| --- \| \| 6.10 \| \| 0.25 \| \| 0.50 \| |
| *Mean ±SE (n=4)* | *3.08 ± 0.76%* | *3.45 ± 1.78%* |

*(g) M/s. Oceanic Institute, Hawaii* (Transit duration-75 hrs) during the period 2009-2012

| *Total brooders supplied* | *Transit Mortality (%)* | *Quarantine Mortality (%)* |
| --- | --- | --- |
| 7260 | \| 2.00 \| \| --- \| \| 2.36 \| \| 0.90 \| \| 2.12 \| \| 2.64 \| \| 2.17 \| \| 0.95 \| \| 5.60 \| \| 1.07 \| | \| 0.18 \| \| --- \| \| 2.23 \| \| 3.63 \| \| 2.80 \| \| 1.48 \| \| 0.50 \| \| 0.36 \| \| 4.48 \| \| 4.28 \| |
|  |  |  |
| *Mean ±SE (n=9)* | *2.201 ± 0.47%* | *2.22 ± 0.0.56%* |

*(h) M/s. Shrimp Improvement systems (SIS), Hawaii* (Transit duration-75 hrs) during the period 2015-2016

| *Total brooders supplied* | *Transit Mortality (%)* | *Quarantine Mortality (%)* |
| --- | --- | --- |
| 4800 | \| 4.25 \| \| --- \| \| 16.00 \| \| 5.00 \| \| 1.50 \| \| 2.50 \| \| 3.00 \| \| 2.50 \| \| 2.25 \| \| 3.00 \| \| 7.00 \| | \| 10.19 \| \| --- \| \| 40.85 \| \| 0.25 \| \| 3.63 \| \| 16.62 \| \| 2.83 \| \| 5.54 \| \| 9.59 \| \| 6.38 \| \| 1.14 \| |
|  |  |  |
| *Mean ± SE (n=10)* | 2.20 ± 0.47% | 9.70 ± 3.79% |

*(i) M/s. Kona Bay Marine Resources, Hawaii* (Transit duration-75 hrs) during the period 2009-2016

| *Total brooders supplied* | *Transit Mortality (%)* | *Quarantine Mortality (%)* |
| --- | --- | --- |
| 106044 | \| 1.80 \| \| --- \| \| 1.56 \| \| 1.79 \| \| 12.95 \| \| 3.64 \| \| 1.63 \| \| 4.00 \| \| 7.88 \| \| 1.17 \| \| 5.38 \| \| 8.38 \| \| 7.75 \| \| 3.25 \| \| 5.00 \| \| 6.50 \| \| 7.50 \| \| 8.00 \| \| 3.33 \| \| 4.25 \| \| 5.00 \| \| 6.00 \| \| 1.75 \| \| 1.00 \| \| 2.75 \| \| 2.50 \| \| 1.38 \| \| 2.50 \| \| 4.00 \| \| 2.13 \| \| 2.00 \| \| 2.25 \| \| 4.13 \| \| 3.38 \| \| 6.50 \| \| 5.50 \| \| 4.25 \| \| 4.00 \| \| 5.00 \| \| 2.50 \| \| 2.50 \| \| 2.58 \| \| 3.75 \| \| 3.00 \| \| 2.75 \| \| 5.00 \| \| 2.75 \| \| 2.67 \| \| 2.75 \| \| 7.50 \| \| 2.88 \| \| 5.50 \| \| 2.63 \| \| 3.75 \| \| 3.00 \| \| 4.75 \| \| 3.13 \| \| 3.25 \| \| 1.63 \| \| 2.50 \| \| 2.88 \| \| 2.25 \| \| 3.00 \| \| 5.75 \| \| 3.50 \| \| 2.62 \| \| 2.25 \| \| 1.88 \| \| 1.58 \| \| 2.08 \| \| 1.75 \| \| 1.63 \| \| 1.75 \| \| 2.50 \| \| 6.50 \| \| 5.00 \| \| 6.88 \| \| 6.25 \| \| 10.50 \| \| 7.50 \| \| 18.08 \| \| 12.17 \| \| 2.50 \| \| 7.00 \| \| 6.25 \| \| 3.00 \| \| 3.50 \| \| 6.13 \| \| 4.40 \| \| 10.06 \| \| 3.75 \| \| 7.50 \| \| 5.00 \| \| 5.00 \| \| 4.13 \| \| 5.00 \| \| 4.25 \| \| 2.25 \| \| 3.50 \| \| 3.91 \| \| 2.25 \| \| 4.25 \| \| 5.25 \| \| 3.38 \| \| 15.00 \| \| 7.00 \| \| 2.50 \| \| 2.38 \| \| 4.88 \| \| 1.33 \| \| 3.20 \| \| 4.00 \| \| 2.75 \| \| 3.75 \| \| 2.90 \| \| 2.50 \| \| 2.83 \| \| 2.17 \| \| 1.63 \| \| 3.00 \| \| 2.25 \| \| 3.67 \| \| 2.50 \| \| 3.75 \| \| 3.38 \| \| 4.00 \| \| 3.83 \| \| 3.42 \| \| 4.75 \| \| 2.88 \| \| 2.75 \| \| 2.15 \| \| 3.00 \| \| 2.32 \| \| 2.38 \| \| 2.00 \| \| 3.00 \| \| 2.38 \| \| 4.17 \| \| 2.30 \| \| 3.00 \| \| 2.50 \| \| 3.00 \| \| 2.50 \| \| 2.86 \| \| 2.25 \| \| 3.50 \| \| 2.00 \| \| 3.25 \| \| 2.50 \| \| 5.47 \| \| 2.17 \| \| 3.27 \| \| 2.33 \| \| 4.25 \| \| 2.13 \| \| 2.50 \| | \| 30.59 \| \| --- \| \| 4.92 \| \| 0.00 \| \| 3.04 \| \| 7.06 \| \| 1.39 \| \| 2.96 \| \| 0.13 \| \| 1.18 \| \| 8.11 \| \| 0.13 \| \| 1.39 \| \| 2.83 \| \| 5.26 \| \| 12.68 \| \| 2.04 \| \| 4.17 \| \| 3.64 \| \| 1.52 \| \| 1.52 \| \| 0.25 \| \| 0.25 \| \| 0.25 \| \| 1.78 \| \| 2.04 \| \| 3.19 \| \| 2.04 \| \| 1.01 \| \| 3.36 \| \| 3.90 \| \| 5.40 \| \| 8.11 \| \| 1.91 \| \| 10.80 \| \| 6.67 \| \| 8.84 \| \| 3.63 \| \| 3.36 \| \| 7.53 \| \| 6.10 \| \| 11.84 \| \| 12.99 \| \| 6.81 \| \| 19.05 \| \| 3.63 \| \| 4.71 \| \| 14.07 \| \| 1.52 \| \| 1.78 \| \| 6.38 \| \| 8.40 \| \| 6.81 \| \| 9.14 \| \| 3.63 \| \| 3.09 \| \| 8.84 \| \| 6.95 \| \| 10.80 \| \| 3.36 \| \| 3.76 \| \| 2.30 \| \| 2.56 \| \| 8.40 \| \| 0.25 \| \| 0.15 \| \| 0.25 \| \| 0.69 \| \| 1.10 \| \| 0.17 \| \| 1.52 \| \| 0.76 \| \| 4.17 \| \| 2.04 \| \| 7.10 \| \| 14.29 \| \| 11.89 \| \| 4.71 \| \| 3.99 \| \| 9.29 \| \| 14.72 \| \| 10.50 \| \| 5.54 \| \| 16.96 \| \| 17.53 \| \| 1.78 \| \| 3.90 \| \| 11.27 \| \| 13.12 \| \| 33.14 \| \| 17.99 \| \| 8.11 \| \| 10.34 \| \| 16.96 \| \| 11.27 \| \| 12.68 \| \| 18.69 \| \| 16.28 \| \| 9.89 \| \| 9.03 \| \| 11.11 \| \| 7.24 \| \| 4.44 \| \| 3.36 \| \| 4.44 \| \| 6.67 \| \| 5.82 \| \| 6.67 \| \| 14.12 \| \| 13.71 \| \| 11.61 \| \| 2.56 \| \| 5.82 \| \| 10.50 \| \| 5.26 \| \| 5.54 \| \| 2.74 \| \| 2.30 \| \| 3.90 \| \| 3.63 \| \| 9.44 \| \| 11.84 \| \| 8.11 \| \| 6.24 \| \| 4.17 \| \| 6.38 \| \| 5.63 \| \| 6.86 \| \| 12.99 \| \| 4.71 \| \| 2.56 \| \| 1.51 \| \| 2.56 \| \| 3.60 \| \| 3.49 \| \| 2.70 \| \| 1.78 \| \| 1.78 \| \| 3.81 \| \| 1.10 \| \| 1.01 \| \| 1.12 \| \| 8.70 \| \| 5.54 \| \| 5.07 \| \| 2.04 \| \| 2.30 \| \| 6.10 \| \| 2.43 \| \| 3.99 \| \| 23.97 \| \| 11.52 \| \| 6.53 \| \| 3.27 \| \| 0.25 \| \| 2.17 \| \| 1.01 \| |
|  |  |  |
| *Mean ± SE (n=156)* | *3.94 ± 0.20%* | *6.27 ± 0.45%* |

*(j) M/s. Shrimp Improvement systems (SIS), Singapore* (Transit duration-14 hrs) during the period 2010-2016

| *Total brooders supplied* | *Transit Mortality (%)* | *Quarantine Mortality (%)* |
| --- | --- | --- |
| 6850 | \| 0.36 \| \| --- \| \| 1.05 \| \| 0.20 \| \| 0.36 \| \| 0.36 \| \| 1.84 \| \| 0.89 \| \| 1.50 \| \| 0.63 \| \| 1.25 \| \| 0.94 \| \| 1.75 \| | \| 0.36 \| \| --- \| \| 0.00 \| \| 1.01 \| \| 0.54 \| \| 0.72 \| \| 0.13 \| \| 0.00 \| \| 2.83 \| \| 0.88 \| \| 0.00 \| \| 0.16 \| \| 0.88 \| |
|  |  |  |
| *Mean ±SE (n=12)* | *0.93±0.0.16%* | *0.63±0.0.23%* |

*(k) M/s. Shrimp Improvement systems (SIS), Florida* (Transit duration-38 hrs) during the period 2009-2016

| *Total brooders supplied* | *Transit Mortality (%)* | *Quarantine Mortality (%)* |
| --- | --- | --- |
| 581954 | \| 0.73 \| \| --- \| \| 0.00 \| \| 1.07 \| \| 1.30 \| \| 4.18 \| \| 2.88 \| \| 1.34 \| \| 1.44 \| \| 2.94 \| \| 1.79 \| \| 2.75 \| \| 1.14 \| \| 1.50 \| \| 2.67 \| \| 4.08 \| \| 3.21 \| \| 1.07 \| \| 5.00 \| \| 0.71 \| \| 1.33 \| \| 2.00 \| \| 4.29 \| \| 7.00 \| \| 1.61 \| \| 1.79 \| \| 1.00 \| \| 0.71 \| \| 1.07 \| \| 1.25 \| \| 0.42 \| \| 0.00 \| \| 1.25 \| \| 1.00 \| \| 0.00 \| \| 0.00 \| \| 0.79 \| \| 2.11 \| \| 3.53 \| \| 6.15 \| \| 1.60 \| \| 0.95 \| \| 2.14 \| \| 5.36 \| \| 0.71 \| \| 0.71 \| \| 1.07 \| \| 1.96 \| \| 1.96 \| \| 0.71 \| \| 2.32 \| \| 0.71 \| \| 1.07 \| \| 0.54 \| \| 2.14 \| \| 4.29 \| \| 4.40 \| \| 1.07 \| \| 2.14 \| \| 1.07 \| \| 3.57 \| \| 0.36 \| \| 1.43 \| \| 0.89 \| \| 0.54 \| \| 2.32 \| \| 0.54 \| \| 2.86 \| \| 0.89 \| \| 0.00 \| \| 1.43 \| \| 3.93 \| \| 3.21 \| \| 2.14 \| \| 1.07 \| \| 0.36 \| \| 1.07 \| \| 0.71 \| \| 1.43 \| \| 0.71 \| \| 1.43 \| \| 1.07 \| \| 0.71 \| \| 1.07 \| \| 0.71 \| \| 0.71 \| \| 1.07 \| \| 0.71 \| \| 0.71 \| \| 0.89 \| \| 0.71 \| \| 1.07 \| \| 1.79 \| \| 1.07 \| \| 1.43 \| \| 1.79 \| \| 1.43 \| \| 1.43 \| \| 1.79 \| \| 3.57 \| \| 1.07 \| \| 0.71 \| \| 1.07 \| \| 1.07 \| \| 1.07 \| \| 0.71 \| \| 1.79 \| \| 1.43 \| \| 1.43 \| \| 0.89 \| \| 1.25 \| \| 0.89 \| \| 0.71 \| \| 0.71 \| \| 0.71 \| \| 1.07 \| \| 1.79 \| \| 3.57 \| \| 1.25 \| \| 1.43 \| \| 1.07 \| \| 1.07 \| \| 0.89 \| \| 1.79 \| \| 1.07 \| \| 0.71 \| \| 1.43 \| \| 1.07 \| \| 0.71 \| \| 1.43 \| \| 1.43 \| \| 0.71 \| \| 1.07 \| \| 2.14 \| \| 1.43 \| \| 0.71 \| \| 2.50 \| \| 2.37 \| \| 1.17 \| \| 1.39 \| \| 3.21 \| \| 1.43 \| \| 1.18 \| \| 0.79 \| \| 1.43 \| \| 2.50 \| \| 0.97 \| \| 0.76 \| \| 1.62 \| \| 1.43 \| \| 1.84 \| \| 1.08 \| \| 1.08 \| \| 1.27 \| \| 1.15 \| \| 1.55 \| \| 1.45 \| \| 1.45 \| \| 1.50 \| \| 1.18 \| \| 3.25 \| \| 1.25 \| \| 1.25 \| \| 1.58 \| \| 1.13 \| \| 2.63 \| \| 2.25 \| \| 1.43 \| \| 1.25 \| \| 1.50 \| \| 1.75 \| \| 1.67 \| \| 2.00 \| \| 1.75 \| \| 1.79 \| \| 1.84 \| \| 1.50 \| \| 1.50 \| \| 1.25 \| \| 2.75 \| \| 2.37 \| \| 1.32 \| \| 3.33 \| \| 5.00 \| \| 2.78 \| \| 1.39 \| \| 2.13 \| \| 3.13 \| \| 2.75 \| \| 2.50 \| \| 2.88 \| \| 2.50 \| \| 1.50 \| \| 4.72 \| \| 3.00 \| \| 3.33 \| \| 1.39 \| \| 1.32 \| \| 2.63 \| \| 1.05 \| \| 1.39 \| \| 1.25 \| \| 1.75 \| \| 1.75 \| \| 1.75 \| \| 1.75 \| \| 1.00 \| \| 1.25 \| \| 1.25 \| \| 1.25 \| \| 1.25 \| \| 1.50 \| \| 1.00 \| \| 1.67 \| \| 1.58 \| \| 1.84 \| \| 1.75 \| \| 1.25 \| \| 1.25 \| \| 1.50 \| \| 2.50 \| \| 2.11 \| \| 3.75 \| \| 1.75 \| \| 2.14 \| \| 1.75 \| \| 1.88 \| \| 2.50 \| \| 1.04 \| \| 1.00 \| \| 2.75 \| \| 2.50 \| \| 2.50 \| \| 2.50 \| \| 1.75 \| \| 1.84 \| \| 1.39 \| \| 1.50 \| \| 1.46 \| \| 1.25 \| \| 1.45 \| \| 1.75 \| \| 2.50 \| \| 1.88 \| \| 3.33 \| \| 3.00 \| \| 2.50 \| \| 2.75 \| \| 4.17 \| \| 2.25 \| \| 3.50 \| \| 3.00 \| \| 1.50 \| \| 1.50 \| \| 2.00 \| \| 1.75 \| \| 1.50 \| \| 2.13 \| \| 4.00 \| \| 1.58 \| \| 5.00 \| \| 1.50 \| \| 3.68 \| \| 2.00 \| \| 1.50 \| \| 0.28 \| \| 1.07 \| \| 1.25 \| \| 1.25 \| \| 1.25 \| \| 1.11 \| \| 0.50 \| \| 1.25 \| \| 1.00 \| \| 0.75 \| \| 0.94 \| \| 0.75 \| \| 1.25 \| \| 1.00 \| \| 1.00 \| \| 1.50 \| \| 1.00 \| \| 1.75 \| \| 1.00 \| \| 3.25 \| \| 2.22 \| \| 3.38 \| \| 4.88 \| \| 3.95 \| \| 3.38 \| \| 2.50 \| \| 1.25 \| \| 1.50 \| \| 1.25 \| \| 1.21 \| \| 1.05 \| \| 1.25 \| \| 1.25 \| \| 2.63 \| \| 2.31 \| \| 1.25 \| \| 1.32 \| \| 1.38 \| \| 1.50 \| \| 1.50 \| \| 1.50 \| \| 1.50 \| \| 2.00 \| \| 1.32 \| \| 2.11 \| \| 1.58 \| \| 0.63 \| \| 1.75 \| \| 1.25 \| \| 1.35 \| \| 0.88 \| \| 2.50 \| \| 1.50 \| \| 1.00 \| \| 1.96 \| \| 1.94 \| \| 1.46 \| \| 1.00 \| \| 0.92 \| \| 0.96 \| \| 1.00 \| \| 1.00 \| \| 1.75 \| \| 1.00 \| \| 1.25 \| \| 1.28 \| \| 1.25 \| \| 1.50 \| \| 1.11 \| \| 1.00 \| \| 0.88 \| \| 0.95 \| \| 1.00 \| \| 0.25 \| \| 0.83 \| \| 0.88 \| \| 1.00 \| \| 1.39 \| \| 1.25 \| \| 1.25 \| \| 1.11 \| \| 1.25 \| \| 1.14 \| \| 1.43 \| \| 1.25 \| \| 1.50 \| \| 0.79 \| \| 3.00 \| \| 1.28 \| \| 1.58 \| \| 1.50 \| \| 3.33 \| \| 3.50 \| \| 2.25 \| \| 1.72 \| \| 1.67 \| \| 1.32 \| \| 1.25 \| \| 0.88 \| \| 1.32 \| \| 1.58 \| \| 1.62 \| \| 1.58 \| \| 1.25 \| \| 1.25 \| \| 1.50 \| \| 1.50 \| \| 1.25 \| \| 2.60 \| \| 1.84 \| \| 3.33 \| \| 2.50 \| \| 1.75 \| \| 1.13 \| \| 1.50 \| \| 1.94 \| \| 2.22 \| \| 2.78 \| \| 2.25 \| \| 2.86 \| \| 1.63 \| \| 3.03 \| \| 1.84 \| \| 3.33 \| \| 3.00 \| \| 1.88 \| \| 3.13 \| \| 2.00 \| \| 2.24 \| \| 2.24 \| \| 2.25 \| \| 2.37 \| \| 1.63 \| \| 2.63 \| \| 1.58 \| \| 2.08 \| \| 2.89 \| \| 4.17 \| \| 2.50 \| \| 2.14 \| \| 2.11 \| \| 3.13 \| \| 1.84 \| \| 2.63 \| \| 2.11 \| \| 2.78 \| \| 1.50 \| \| 1.75 \| \| 2.89 \| \| 2.11 \| \| 2.13 \| \| 2.76 \| \| 2.37 \| \| 3.57 \| \| 2.11 \| \| 2.89 \| \| 1.04 \| \| 3.42 \| \| 1.84 \| \| 2.50 \| \| 1.58 \| \| 2.22 \| \| 2.08 \| \| 2.50 \| \| 1.97 \| \| 1.56 \| \| 1.75 \| \| 3.04 \| \| 1.94 \| \| 2.00 \| \| 1.58 \| \| 1.32 \| \| 2.11 \| \| 1.32 \| \| 1.93 \| \| 3.16 \| \| 1.32 \| \| 1.84 \| \| 1.84 \| \| 1.84 \| \| 1.18 \| \| 2.76 \| \| 1.32 \| \| 1.32 \| \| 1.32 \| \| 1.05 \| \| 1.71 \| \| 2.86 \| \| 1.84 \| \| 1.45 \| \| 1.58 \| \| 1.32 \| \| 1.14 \| \| 1.45 \| \| 1.58 \| \| 1.58 \| \| 2.76 \| \| 1.71 \| \| 1.71 \| \| 2.63 \| \| 1.71 \| \| 1.84 \| \| 1.93 \| \| 2.11 \| \| 1.32 \| \| 1.32 \| \| 1.97 \| \| 1.84 \| \| 3.68 \| \| 1.64 \| \| 1.71 \| \| 1.58 \| \| 1.84 \| \| 1.84 \| \| 2.63 \| \| 1.43 \| \| 1.84 \| \| 1.83 \| \| 1.05 \| \| 2.81 \| \| 1.58 \| \| 2.35 \| \| 2.54 \| \| 1.58 \| \| 1.84 \| \| 1.70 \| \| 3.16 \| \| 2.19 \| \| 2.11 \| \| 2.22 \| \| 1.67 \| \| 1.84 \| \| 2.37 \| \| 2.37 \| \| 2.11 \| \| 1.58 \| \| 2.37 \| \| 1.71 \| \| 2.89 \| \| 2.24 \| \| 2.22 \| \| 3.61 \| \| 2.11 \| \| 2.33 \| \| 3.16 \| \| 3.68 \| \| 2.50 \| \| 6.58 \| \| 2.89 \| \| 2.89 \| \| 2.22 \| \| 2.08 \| \| 3.68 \| \| 2.89 \| \| 3.06 \| \| 2.33 \| \| 3.89 \| \| 4.46 \| \| 3.61 \| \| 3.06 \| \| 3.95 \| \| 2.89 \| \| 2.50 \| \| 2.63 \| \| 4.47 \| \| 2.37 \| \| 3.82 \| \| 2.11 \| \| 1.84 \| \| 1.45 \| \| 2.63 \| \| 2.37 \| \| 2.37 \| \| 3.16 \| \| 2.76 \| \| 3.95 \| \| 2.89 \| \| 4.21 \| \| 7.89 \| \| 2.59 \| \| 3.68 \| \| 1.84 \| \| 3.09 \| \| 2.98 \| \| 4.44 \| \| 2.37 \| \| 1.32 \| \| 2.08 \| \| 8.77 \| \| 3.03 \| \| 2.37 \| \| 2.37 \| \| 1.45 \| \| 2.27 \| \| 2.11 \| \| 1.49 \| \| 1.94 \| \| 1.71 \| \| 1.58 \| \| 2.11 \| \| 1.39 \| \| 1.67 \| \| 1.84 \| \| 1.58 \| \| 1.32 \| \| 1.58 \| \| 2.63 \| \| 1.05 \| \| 1.32 \| \| 2.11 \| \| 1.71 \| \| 2.00 \| \| 1.32 \| \| 1.67 \| \| 1.49 \| \| 1.58 \| \| 2.11 \| \| 1.32 \| \| 2.37 \| \| 1.32 \| \| 1.45 \| \| 2.63 \| \| 2.11 \| \| 2.03 \| \| 1.03 \| \| 1.67 \| \| 1.61 \| \| 1.18 \| \| 1.97 \| \| 2.24 \| \| 2.89 \| \| 1.18 \| \| 1.58 \| \| 1.84 \| \| 1.12 \| \| 1.84 \| \| 2.11 \| \| 1.45 \| \| 0.79 \| \| 1.94 \| \| 1.84 \| \| 0.86 \| \| 1.84 \| \| 1.05 \| \| 1.58 \| \| 0.66 \| \| 2.00 \| \| 0.79 \| \| 1.18 \| \| 1.58 \| \| 3.03 \| \| 3.95 \| \| 3.68 \| \| 2.37 \| \| 1.05 \| \| 1.05 \| \| 1.05 \| \| 1.32 \| \| 1.32 \| \| 1.58 \| \| 0.88 \| \| 1.05 \| \| 1.32 \| \| 1.32 \| \| 1.33 \| \| 0.92 \| \| 0.79 \| \| 1.37 \| \| 1.52 \| \| 1.18 \| \| 1.18 \| \| 1.05 \| \| 1.32 \| \| 0.70 \| \| 0.96 \| \| 0.79 \| \| 1.05 \| \| 1.25 \| \| 0.92 \| \| 1.05 \| \| 1.11 \| \| 0.79 \| \| 0.53 \| \| 0.58 \| \| 0.66 \| \| 0.53 \| \| 0.92 \| \| 0.83 \| \| 1.05 \| \| 0.53 \| \| 0.56 \| \| 0.66 \| \| 0.75 \| \| 0.83 \| \| 1.18 \| \| 1.32 \| \| 6.36 \| \| 0.94 \| \| 1.45 \| \| 0.66 \| \| 1.39 \| \| 1.11 \| \| 0.66 \| \| 1.32 \| \| 1.84 \| \| 1.71 \| \| 1.59 \| \| 1.32 \| \| 1.39 \| \| 1.18 \| \| 1.76 \| \| 0.79 \| \| 1.05 \| \| 1.18 \| \| 1.18 \| \| 1.50 \| \| 1.84 \| \| 1.05 \| \| 0.79 \| \| 1.32 \| \| 1.71 \| \| 0.79 \| \| 1.05 \| \| 0.92 \| \| 1.11 \| \| 1.05 \| \| 0.92 \| \| 1.05 \| \| 1.33 \| \| 1.39 \| \| 1.11 \| \| 1.18 \| \| 0.66 \| \| 1.05 \| \| 0.88 \| \| 1.05 \| \| 1.09 \| \| 1.25 \| \| 1.18 \| \| 1.00 \| \| 0.79 \| \| 1.21 \| \| 0.66 \| \| 0.79 \| \| 1.11 \| \| 0.67 \| \| 1.36 \| \| 1.25 \| \| 1.04 \| \| 0.79 \| \| 0.96 \| \| 1.05 \| \| 1.25 \| \| 1.18 \| \| 0.94 \| \| 1.53 \| \| 1.25 \| \| 1.00 \| \| 1.11 \| \| 1.58 \| \| 0.92 \| \| 1.67 \| \| 0.88 \| \| 1.58 \| \| 0.79 \| \| 0.92 \| \| 0.69 \| \| 1.94 \| \| 1.05 \| \| 1.42 \| \| 1.44 \| \| 0.92 \| \| 1.21 \| \| 1.11 \| \| 1.75 \| \| 1.88 \| \| 1.13 \| \| 1.84 \| \| 1.05 \| \| 0.00 \| \| 1.49 \| \| 1.32 \| \| 1.05 \| \| 0.79 \| \| 1.32 \| \| 1.32 \| \| 2.11 \| \| 0.92 \| \| 1.45 \| \| 3.95 \| \| 1.05 \| \| 2.11 \| \| 1.32 \| \| 0.79 \| \| 1.05 \| \| 1.00 \| \| 1.05 \| \| 0.88 \| \| 1.25 \| \| 1.18 \| \| 1.58 \| \| 1.00 \| \| 1.58 \| \| 1.05 \| \| 1.32 \| \| 1.05 \| \| 2.25 \| \| 0.92 \| \| 1.00 \| \| 1.45 \| \| 1.05 \| \| 1.25 \| \| 1.14 \| \| 0.57 \| \| 1.09 \| \| 0.53 \| \| 1.05 \| \| 0.53 \| \| 0.66 \| \| 0.88 \| \| 1.05 \| \| 0.79 \| \| 1.97 \| \| 1.13 \| \| 1.11 \| \| 1.25 \| \| 0.59 \| \| 1.84 \| \| 1.25 \| \| 1.45 \| \| 1.05 \| \| 0.44 \| \| 0.66 \| \| 0.56 \| \| 0.79 \| \| 0.88 \| \| 0.51 \| \| 0.53 \| \| 0.50 \| \| 0.50 \| \| 0.53 \| \| 0.79 \| \| 0.50 \| \| 0.53 \| \| 1.84 \| \| 0.79 \| \| 0.70 \| \| 1.25 \| \| 0.75 \| \| 0.63 \| \| 1.05 \| \| 0.75 \| \| 1.05 \| \| 0.88 \| \| 1.13 \| \| 1.17 \| \| 1.05 \| \| 1.50 \| \| 1.39 \| \| 1.38 \| \| 1.18 \| \| 3.00 \| \| 0.92 \| \| 0.50 \| \| 0.75 \| \| 0.88 \| \| 1.00 \| \| 1.00 \| \| 1.13 \| \| 1.50 \| \| 1.00 \| \| 2.00 \| \| 0.67 \| \| 1.05 \| \| 1.32 \| \| 0.90 \| \| 0.64 \| \| 0.87 \| \| 1.45 \| \| 1.05 \| \| 1.32 \| \| 1.25 \| \| 1.25 \| \| 0.81 \| \| 0.50 \| \| 0.63 \| \| 0.91 \| \| 0.88 \| \| 1.67 \| \| 1.75 \| \| 2.63 \| \| 0.75 \| \| 2.25 \| \| 1.63 \| \| 1.38 \| \| 2.75 \| \| 1.50 \| \| 1.82 \| \| 1.00 \| \| 1.50 \| \| 1.67 \| \| 1.25 \| \| 1.08 \| \| 0.88 \| \| 0.92 \| \| 1.67 \| \| 1.67 \| \| 1.92 \| \| 1.25 \| \| 2.00 \| \| 2.56 \| \| 1.88 \| \| 4.41 \| \| 1.83 \| \| 2.33 \| \| 3.25 \| \| 3.25 \| \| 2.75 \| \| 2.13 \| \| 1.25 \| \| 2.25 \| \| 1.71 \| \| 1.31 \| \| 2.25 \| \| 2.00 \| \| 2.06 \| \| 1.63 \| \| 1.75 \| \| 2.50 \| \| 2.63 \| \| 1.50 \| \| 2.50 \| \| 1.38 \| \| 2.28 \| \| 2.86 \| \| 1.20 \| \| 1.38 \| \| 3.00 \| \| 1.50 \| \| 1.25 \| \| 2.13 \| \| 1.50 \| \| 1.67 \| \| 1.25 \| \| 1.25 \| \| 2.91 \| \| 3.75 \| \| 3.50 \| \| 2.00 \| \| 1.25 \| \| 1.38 \| \| 1.54 \| \| 2.00 \| \| 0.85 \| \| 5.50 \| \| 2.38 \| \| 3.00 \| \| 3.75 \| \| 4.00 \| \| 3.00 \| \| 4.88 \| \| 1.33 \| \| 2.50 \| \| 5.75 \| \| 3.00 \| \| 2.83 \| \| 2.00 \| \| 2.00 \| \| 1.50 \| \| 3.17 \| \| 2.50 \| \| 1.50 \| \| 2.50 \| \| 2.50 \| \| 2.78 \| \| 1.94 \| \| 2.00 \| \| 1.17 \| \| 2.88 \| \| 2.94 \| \| 3.50 \| \| 5.00 \| \| 2.50 \| \| 4.00 \| \| 4.25 \| \| 3.75 \| \| 2.00 \| \| 4.17 \| \| 4.38 \| \| 3.50 \| \| 2.75 \| \| 3.00 \| \| 2.13 \| \| 1.83 \| \| 1.50 \| \| 1.38 \| \| 2.25 \| \| 2.00 \| \| 1.75 \| \| 1.25 \| \| 1.00 \| \| 1.13 \| \| 0.88 \| \| 1.60 \| \| 2.00 \| \| 1.83 \| \| 1.31 \| \| 1.88 \| \| 1.75 \| \| 2.25 \| \| 2.08 \| \| 1.83 \| \| 2.08 \| \| 2.25 \| \| 1.75 \| \| 1.30 \| \| 2.25 \| \| 1.67 \| \| 2.17 \| \| 2.75 \| \| 2.75 \| \| 4.50 \| \| 1.42 \| \| 2.67 \| \| 3.25 \| \| 4.25 \| \| 1.63 \| \| 2.50 \| \| 1.67 \| \| 3.25 \| \| 3.67 \| \| 3.00 \| \| 2.00 \| \| 2.38 \| \| 4.50 \| \| 2.00 \| \| 4.50 \| \| 1.83 \| \| 2.63 \| \| 2.38 \| \| 3.25 \| \| 2.50 \| | \| 3.38 \| \| --- \| \| 0.00 \| \| 8.53 \| \| 3.14 \| \| 8.91 \| \| 5.70 \| \| 7.45 \| \| 6.51 \| \| 6.25 \| \| 12.00 \| \| 3.96 \| \| 6.54 \| \| 6.10 \| \| 6.38 \| \| 4.26 \| \| 3.32 \| \| 6.87 \| \| 5.26 \| \| 9.80 \| \| 7.91 \| \| 2.04 \| \| 0.00 \| \| 6.95 \| \| 1.27 \| \| 4.67 \| \| 2.46 \| \| 3.58 \| \| 1.08 \| \| 3.51 \| \| 3.00 \| \| 2.81 \| \| 1.08 \| \| 0.81 \| \| 2.53 \| \| 0.61 \| \| 1.33 \| \| 2.70 \| \| 4.94 \| \| 28.08 \| \| 6.84 \| \| 6.60 \| \| 5.07 \| \| 14.29 \| \| 3.32 \| \| 3.32 \| \| 1.08 \| \| 3.90 \| \| 3.51 \| \| 3.90 \| \| 6.46 \| \| 13.82 \| \| 5.66 \| \| 4.67 \| \| 4.28 \| \| 8.95 \| \| 10.24 \| \| 6.87 \| \| 5.26 \| \| 4.09 \| \| 4.48 \| \| 2.00 \| \| 1.08 \| \| 3.90 \| \| 1.08 \| \| 6.26 \| \| 1.82 \| \| 5.26 \| \| 1.63 \| \| 2.19 \| \| 2.56 \| \| 3.32 \| \| 4.09 \| \| 4.48 \| \| 0.72 \| \| 3.32 \| \| 1.82 \| \| 0.72 \| \| 0.36 \| \| 4.67 \| \| 2.56 \| \| 2.56 \| \| 2.56 \| \| 1.45 \| \| 0.72 \| \| 1.45 \| \| 1.63 \| \| 2.00 \| \| 2.38 \| \| 0.18 \| \| 3.13 \| \| 3.70 \| \| 1.45 \| \| 1.08 \| \| 1.82 \| \| 1.08 \| \| 1.45 \| \| 2.19 \| \| 5.26 \| \| 100.00 \| \| 1.08 \| \| 0.36 \| \| 0.72 \| \| 1.08 \| \| 0.00 \| \| 2.94 \| \| 2.56 \| \| 1.45 \| \| 0.00 \| \| 1.45 \| \| 0.00 \| \| 2.00 \| \| 1.45 \| \| 1.08 \| \| 3.13 \| \| 2.38 \| \| 1.82 \| \| 5.66 \| \| 1.08 \| \| 0.00 \| \| 0.00 \| \| 1.82 \| \| 2.56 \| \| 2.56 \| \| 0.36 \| \| 1.08 \| \| 4.48 \| \| 1.82 \| \| 3.70 \| \| 0.36 \| \| 3.51 \| \| 1.08 \| \| 0.72 \| \| 0.36 \| \| 1.08 \| \| 2.94 \| \| 1.82 \| \| 2.01 \| \| 3.81 \| \| 0.28 \| \| 0.00 \| \| 0.00 \| \| 0.93 \| \| 2.70 \| \| 2.94 \| \| 2.94 \| \| 2.42 \| \| 1.54 \| \| 3.06 \| \| 5.26 \| \| 4.54 \| \| 0.00 \| \| 0.27 \| \| 1.54 \| \| 1.43 \| \| 1.33 \| \| 0.26 \| \| 1.88 \| \| 0.25 \| \| 4.54 \| \| 1.78 \| \| 2.30 \| \| 0.25 \| \| 2.29 \| \| 0.50 \| \| 3.76 \| \| 0.50 \| \| 1.82 \| \| 3.90 \| \| 4.44 \| \| 1.52 \| \| 1.12 \| \| 4.17 \| \| 6.95 \| \| 1.82 \| \| 4.11 \| \| 4.44 \| \| 0.00 \| \| 1.78 \| \| 1.27 \| \| 0.00 \| \| 0.80 \| \| 1.69 \| \| 2.83 \| \| 0.00 \| \| 0.84 \| \| 2.96 \| \| 5.54 \| \| 0.76 \| \| 2.30 \| \| 1.27 \| \| 0.25 \| \| 0.76 \| \| 3.45 \| \| 2.83 \| \| 1.98 \| \| 1.41 \| \| 2.70 \| \| 1.60 \| \| 3.54 \| \| 1.41 \| \| 2.04 \| \| 3.90 \| \| 1.14 \| \| 3.63 \| \| 3.63 \| \| 1.27 \| \| 2.56 \| \| 1.27 \| \| 0.50 \| \| 0.76 \| \| 2.04 \| \| 3.90 \| \| 1.69 \| \| 1.33 \| \| 0.00 \| \| 0.00 \| \| 0.76 \| \| 0.25 \| \| 2.56 \| \| 4.71 \| \| 3.26 \| \| 5.26 \| \| 1.78 \| \| 4.09 \| \| 3.09 \| \| 4.17 \| \| 6.81 \| \| 1.27 \| \| 3.90 \| \| 0.50 \| \| 0.25 \| \| 0.13 \| \| 3.36 \| \| 3.63 \| \| 2.84 \| \| 2.13 \| \| 3.63 \| \| 0.42 \| \| 2.56 \| \| 5.70 \| \| 12.68 \| \| 16.96 \| \| 11.58 \| \| 4.05 \| \| 0.00 \| \| 0.00 \| \| 0.00 \| \| 0.42 \| \| 0.50 \| \| 0.25 \| \| 0.00 \| \| 1.01 \| \| 1.01 \| \| 1.65 \| \| 3.09 \| \| 3.36 \| \| 1.91 \| \| 0.00 \| \| 0.40 \| \| 0.25 \| \| 0.00 \| \| 0.00 \| \| 0.00 \| \| 0.25 \| \| 1.73 \| \| 0.00 \| \| 0.00 \| \| 0.00 \| \| 0.00 \| \| 0.00 \| \| 0.00 \| \| 3.63 \| \| 0.00 \| \| 0.00 \| \| 0.31 \| \| 1.01 \| \| 1.52 \| \| 0.00 \| \| 1.52 \| \| 0.50 \| \| 0.76 \| \| 0.76 \| \| 0.00 \| \| 2.30 \| \| 1.69 \| \| 1.78 \| \| 2.56 \| \| 0.00 \| \| 1.27 \| \| 0.00 \| \| 0.00 \| \| 0.50 \| \| 0.00 \| \| 2.47 \| \| 1.33 \| \| 6.10 \| \| 3.09 \| \| 1.60 \| \| 0.00 \| \| 0.63 \| \| 0.26 \| \| 1.52 \| \| 1.91 \| \| 2.83 \| \| 1.01 \| \| 2.56 \| \| 0.00 \| \| 0.00 \| \| 0.26 \| \| 0.00 \| \| 0.25 \| \| 0.25 \| \| 2.43 \| \| 1.65 \| \| 0.88 \| \| 1.78 \| \| 1.52 \| \| 1.01 \| \| 0.54 \| \| 0.00 \| \| 0.00 \| \| 0.00 \| \| 0.26 \| \| 1.96 \| \| 0.50 \| \| 0.88 \| \| 1.78 \| \| 2.56 \| \| 0.50 \| \| 0.26 \| \| 0.76 \| \| 0.25 \| \| 0.28 \| \| 0.25 \| \| 0.25 \| \| 0.41 \| \| 0.25 \| \| 0.63 \| \| 0.00 \| \| 0.25 \| \| 0.00 \| \| 0.84 \| \| 0.13 \| \| 2.56 \| \| 3.15 \| \| 0.76 \| \| 0.00 \| \| 0.36 \| \| 0.50 \| \| 0.76 \| \| 0.26 \| \| 3.36 \| \| 3.16 \| \| 4.68 \| \| 2.04 \| \| 2.56 \| \| 3.09 \| \| 0.25 \| \| 0.31 \| \| 2.56 \| \| 1.33 \| \| 1.78 \| \| 0.76 \| \| 0.53 \| \| 1.60 \| \| 0.00 \| \| 0.00 \| \| 1.52 \| \| 2.04 \| \| 1.27 \| \| 4.03 \| \| 3.90 \| \| 1.59 \| \| 2.98 \| \| 6.67 \| \| 3.36 \| \| 4.17 \| \| 8.99 \| \| 1.65 \| \| 1.98 \| \| 0.98 \| \| 3.15 \| \| 1.01 \| \| 1.82 \| \| 2.70 \| \| 8.88 \| \| 1.33 \| \| 5.26 \| \| 0.50 \| \| 4.92 \| \| 3.23 \| \| 0.25 \| \| 2.15 \| \| 0.00 \| \| 0.25 \| \| 0.00 \| \| 1.39 \| \| 2.15 \| \| 1.60 \| \| 3.90 \| \| 0.00 \| \| 0.00 \| \| 0.00 \| \| 3.32 \| \| 0.26 \| \| 1.27 \| \| 0.53 \| \| 0.26 \| \| 2.98 \| \| 3.45 \| \| 3.49 \| \| 1.27 \| \| 2.98 \| \| 3.12 \| \| 2.96 \| \| 0.93 \| \| 0.00 \| \| 2.56 \| \| 2.98 \| \| 1.60 \| \| 0.63 \| \| 4.97 \| \| 1.33 \| \| 2.24 \| \| 0.26 \| \| 1.69 \| \| 3.00 \| \| 4.35 \| \| 2.01 \| \| 0.63 \| \| 0.88 \| \| 0.36 \| \| 4.05 \| \| 2.30 \| \| 3.83 \| \| 4.11 \| \| 0.26 \| \| 0.40 \| \| 1.97 \| \| 4.97 \| \| 2.70 \| \| 2.01 \| \| 5.56 \| \| 1.88 \| \| 0.66 \| \| 0.66 \| \| 0.80 \| \| 0.80 \| \| 0.53 \| \| 1.33 \| \| 0.53 \| \| 4.17 \| \| 0.80 \| \| 0.00 \| \| 0.66 \| \| 0.53 \| \| 0.57 \| \| 1.88 \| \| 2.29 \| \| 4.11 \| \| 6.29 \| \| 3.26 \| \| 2.84 \| \| 3.83 \| \| 6.89 \| \| 3.83 \| \| 0.26 \| \| 1.47 \| \| 0.26 \| \| 0.53 \| \| 1.20 \| \| 1.33 \| \| 1.33 \| \| 1.85 \| \| 1.20 \| \| 2.43 \| \| 1.74 \| \| 2.43 \| \| 1.06 \| \| 2.56 \| \| 1.88 \| \| 2.92 \| \| 4.68 \| \| 1.97 \| \| 2.70 \| \| 2.72 \| \| 8.06 \| \| 0.00 \| \| 0.00 \| \| 0.57 \| \| 2.43 \| \| 2.89 \| \| 0.00 \| \| 0.28 \| \| 0.28 \| \| 1.33 \| \| 0.26 \| \| 2.70 \| \| 2.43 \| \| 4.97 \| \| 3.54 \| \| 6.89 \| \| 5.56 \| \| 3.97 \| \| 0.28 \| \| 4.05 \| \| 1.33 \| \| 0.84 \| \| 3.97 \| \| 1.06 \| \| 3.40 \| \| 5.26 \| \| 2.98 \| \| 7.65 \| \| 9.09 \| \| 2.71 \| \| 20.63 \| \| 13.43 \| \| 14.65 \| \| 15.83 \| \| 26.32 \| \| 4.67 \| \| 5.11 \| \| 0.56 \| \| 2.70 \| \| 1.33 \| \| 0.28 \| \| 0.00 \| \| 4.54 \| \| 0.00 \| \| 0.00 \| \| 0.00 \| \| 0.00 \| \| 0.00 \| \| 0.00 \| \| 1.06 \| \| 1.60 \| \| 2.15 \| \| 2.43 \| \| 0.53 \| \| 11.44 \| \| 9.35 \| \| 10.95 \| \| 3.76 \| \| 4.68 \| \| 2.43 \| \| 4.78 \| \| 0.62 \| \| 1.12 \| \| 1.06 \| \| 1.33 \| \| 0.42 \| \| 0.71 \| \| 0.00 \| \| 0.00 \| \| 0.53 \| \| 0.00 \| \| 0.46 \| \| 0.26 \| \| 2.24 \| \| 3.15 \| \| 1.88 \| \| 0.00 \| \| 0.00 \| \| 0.56 \| \| 0.00 \| \| 0.00 \| \| 0.00 \| \| 0.35 \| \| 0.44 \| \| 0.53 \| \| 0.80 \| \| 0.80 \| \| 4.11 \| \| 2.01 \| \| 0.63 \| \| 4.97 \| \| 1.33 \| \| 0.80 \| \| 2.01 \| \| 1.74 \| \| 0.26 \| \| 0.13 \| \| 0.26 \| \| 0.53 \| \| 0.26 \| \| 0.13 \| \| 0.16 \| \| 0.15 \| \| 0.00 \| \| 0.27 \| \| 0.26 \| \| 0.13 \| \| 0.40 \| \| 1.60 \| \| 0.26 \| \| 0.13 \| \| 0.26 \| \| 0.26 \| \| 0.53 \| \| 0.13 \| \| 0.40 \| \| 0.26 \| \| 0.56 \| \| 0.80 \| \| 0.20 \| \| 0.53 \| \| 0.26 \| \| 0.26 \| \| 0.26 \| \| 0.67 \| \| 0.26 \| \| 0.13 \| \| 0.80 \| \| 0.40 \| \| 0.26 \| \| 0.53 \| \| 1.60 \| \| 0.97 \| \| 0.62 \| \| 0.26 \| \| 0.13 \| \| 0.26 \| \| 0.26 \| \| 0.15 \| \| 0.26 \| \| 0.26 \| \| 0.26 \| \| 0.33 \| \| 0.13 \| \| 0.13 \| \| 0.34 \| \| 0.19 \| \| 0.13 \| \| 0.13 \| \| 0.26 \| \| 0.53 \| \| 0.18 \| \| 0.09 \| \| 0.13 \| \| 0.26 \| \| 0.63 \| \| 0.13 \| \| 0.26 \| \| 0.28 \| \| 0.40 \| \| 0.00 \| \| 0.08 \| \| 0.40 \| \| 0.13 \| \| 0.53 \| \| 0.50 \| \| 0.26 \| \| 0.80 \| \| 0.47 \| \| 0.13 \| \| 0.13 \| \| 0.59 \| \| 0.13 \| \| 0.26 \| \| 2.80 \| \| 0.47 \| \| 0.26 \| \| 0.40 \| \| 0.14 \| \| 0.56 \| \| 0.86 \| \| 0.26 \| \| 0.53 \| \| 0.13 \| \| 0.32 \| \| 0.09 \| \| 0.28 \| \| 0.13 \| \| 0.89 \| \| 0.40 \| \| 0.53 \| \| 0.20 \| \| 0.26 \| \| 0.25 \| \| 0.26 \| \| 0.26 \| \| 0.26 \| \| 0.53 \| \| 0.26 \| \| 0.13 \| \| 0.26 \| \| 0.26 \| \| 0.28 \| \| 0.26 \| \| 0.13 \| \| 0.80 \| \| 0.33 \| \| 0.56 \| \| 0.56 \| \| 0.40 \| \| 0.13 \| \| 0.53 \| \| 0.29 \| \| 0.26 \| \| 0.63 \| \| 0.25 \| \| 0.29 \| \| 0.42 \| \| 0.18 \| \| 0.40 \| \| 0.13 \| \| 0.26 \| \| 0.28 \| \| 0.08 \| \| 1.38 \| \| 0.50 \| \| 0.42 \| \| 0.93 \| \| 0.44 \| \| 0.53 \| \| 0.25 \| \| 0.13 \| \| 0.16 \| \| 0.56 \| \| 1.01 \| \| 0.38 \| \| 0.28 \| \| 0.26 \| \| 0.13 \| \| 0.33 \| \| 0.09 \| \| 0.26 \| \| 0.26 \| \| 2.29 \| \| 3.60 \| \| 2.86 \| \| 2.98 \| \| 0.93 \| \| 0.55 \| \| 0.26 \| \| 0.43 \| \| 0.28 \| \| 0.25 \| \| 0.38 \| \| 0.25 \| \| 1.60 \| \| 0.26 \| \| 0.33 \| \| 0.26 \| \| 0.89 \| \| 0.80 \| \| 0.44 \| \| 1.60 \| \| 1.33 \| \| 2.70 \| \| 0.26 \| \| 1.33 \| \| 1.06 \| \| 0.40 \| \| 0.26 \| \| 0.26 \| \| 0.66 \| \| 0.26 \| \| 0.33 \| \| 2.01 \| \| 0.50 \| \| 0.25 \| \| 0.26 \| \| 0.26 \| \| 0.25 \| \| 0.80 \| \| 0.26 \| \| 1.33 \| \| 0.40 \| \| 1.27 \| \| 0.66 \| \| 0.67 \| \| 0.53 \| \| 0.53 \| \| 0.08 \| \| 0.09 \| \| 0.14 \| \| 0.16 \| \| 0.09 \| \| 0.26 \| \| 0.26 \| \| 0.13 \| \| 0.25 \| \| 0.26 \| \| 0.26 \| \| 2.15 \| \| 0.50 \| \| 0.28 \| \| 0.13 \| \| 0.07 \| \| 0.26 \| \| 0.13 \| \| 0.13 \| \| 0.26 \| \| 0.09 \| \| 0.26 \| \| 0.28 \| \| 0.13 \| \| 0.25 \| \| 0.13 \| \| 0.80 \| \| 0.25 \| \| 0.50 \| \| 0.13 \| \| 0.26 \| \| 0.25 \| \| 0.26 \| \| 0.53 \| \| 0.26 \| \| 1.06 \| \| 0.25 \| \| 1.39 \| \| 0.88 \| \| 0.80 \| \| 0.25 \| \| 0.26 \| \| 0.76 \| \| 0.25 \| \| 1.78 \| \| 0.80 \| \| 0.25 \| \| 0.28 \| \| 0.13 \| \| 0.13 \| \| 0.25 \| \| 0.13 \| \| 0.76 \| \| 1.01 \| \| 0.38 \| \| 0.76 \| \| 1.27 \| \| 0.76 \| \| 0.76 \| \| 2.04 \| \| 2.04 \| \| 3.45 \| \| 0.26 \| \| 0.26 \| \| 0.97 \| \| 1.30 \| \| 0.48 \| \| 1.60 \| \| 1.60 \| \| 0.13 \| \| 1.52 \| \| 0.76 \| \| 0.19 \| \| 0.25 \| \| 0.25 \| \| 0.38 \| \| 0.88 \| \| 0.42 \| \| 1.91 \| \| 0.76 \| \| 0.13 \| \| 1.01 \| \| 2.56 \| \| 0.13 \| \| 0.13 \| \| 0.50 \| \| 0.23 \| \| 0.25 \| \| 0.25 \| \| 0.56 \| \| 0.25 \| \| 0.93 \| \| 0.25 \| \| 2.01 \| \| 0.08 \| \| 1.17 \| \| 3.45 \| \| 4.71 \| \| 5.82 \| \| 0.78 \| \| 0.13 \| \| 2.31 \| \| 1.52 \| \| 0.08 \| \| 2.56 \| \| 1.27 \| \| 1.65 \| \| 2.43 \| \| 2.30 \| \| 2.30 \| \| 0.80 \| \| 1.72 \| \| 3.00 \| \| 1.27 \| \| 4.10 \| \| 3.09 \| \| 1.01 \| \| 0.76 \| \| 0.53 \| \| 1.27 \| \| 0.50 \| \| 1.91 \| \| 1.24 \| \| 2.94 \| \| 2.18 \| \| 3.76 \| \| 2.04 \| \| 0.25 \| \| 1.01 \| \| 0.63 \| \| 0.25 \| \| 0.08 \| \| 0.17 \| \| 0.88 \| \| 1.66 \| \| 2.30 \| \| 1.52 \| \| 0.76 \| \| 0.25 \| \| 0.13 \| \| 1.12 \| \| 1.78 \| \| 2.45 \| \| 1.52 \| \| 6.67 \| \| 2.04 \| \| 3.54 \| \| 7.82 \| \| 3.99 \| \| 3.63 \| \| 0.76 \| \| 8.11 \| \| 2.65 \| \| 4.71 \| \| 1.87 \| \| 2.56 \| \| 6.57 \| \| 1.52 \| \| 1.35 \| \| 0.25 \| \| 0.50 \| \| 0.38 \| \| 0.76 \| \| 0.28 \| \| 3.16 \| \| 1.78 \| \| 2.39 \| \| 2.83 \| \| 2.10 \| \| 3.63 \| \| 5.26 \| \| 5.96 \| \| 5.26 \| \| 5.82 \| \| 4.44 \| \| 4.44 \| \| 6.76 \| \| 3.76 \| \| 3.63 \| \| 4.03 \| \| 6.10 \| \| 4.85 \| \| 0.84 \| \| 2.21 \| \| 2.83 \| \| 1.01 \| \| 2.04 \| \| 0.25 \| \| 1.01 \| \| 0.76 \| \| 1.65 \| \| 1.14 \| \| 1.01 \| \| 1.87 \| \| 2.39 \| \| 6.17 \| \| 4.30 \| \| 3.36 \| \| 2.04 \| \| 0.67 \| \| 1.87 \| \| 7.53 \| \| 1.61 \| \| 2.70 \| \| 1.11 \| \| 4.71 \| \| 3.09 \| \| 7.33 \| \| 0.25 \| \| 1.78 \| \| 2.56 \| \| 4.17 \| \| 4.17 \| \| 3.09 \| \| 4.99 \| \| 2.70 \| \| 2.56 \| \| 0.17 \| \| 1.27 \| \| 7.33 \| \| 10.04 \| \| 2.04 \| \| 1.27 \| \| 0.50 \| \| 7.67 \| \| 9.59 \| \| 3.99 \| \| 5.54 \| \| 1.01 \| \| 12.68 \| \| 1.01 \| |
|  |  |  |
| *Mean ± SE (n=1006)* | *1.78 ± 0.0.32%* | *2.03 ± 0.0.13%* |

**Note:** The furnished data is from the period of commencement (18^th^ July 2009) of vannamei importation to India till 16^th^ December2016.
